# Systematic Analysis of *Stay-Green* Genes in Six *Ipomoea* Species Reveals the Evolutionary Dynamics, Carotenoid and Anthocyanin Accumulation, and Stress Responses of Sweet Potato

**DOI:** 10.3390/genes16030266

**Published:** 2025-02-24

**Authors:** Zhidan Zuo, Huihui Ma, Longteng Li, Jialin Qian, Minghui Zhang, Xiang Li, Yeshun Sheng, Yuxin Wang

**Affiliations:** 1 College of Life Sciences, Zaozhuang University, Zaozhuang 277160, China; zhidanzuo@outlook.com (Z.Z.); mahuihui8023@126.com (H.M.); 19506786290@163.com (L.L.); m13053686013@163.com (J.Q.); m19506781279@163.com (M.Z.); 13176135815@163.com (X.L.); 15666546018@163.com (Y.S.); 2 Key Laboratory of Sweet Potato Biology and Biotechnology of Ministry of Agriculture and Rural Affairs, College of Agronomy & Biotechnology, China Agricultural University, Beijing 100193, China

**Keywords:** gene family evolution, hormone crosstalk, phylogenetics, pigment accumulation, stress tolerance

## Abstract

Background/Objectives: Stay-green proteins (SGRs) play a vital role in regulating plant chlorophyll degradation and senescence. However, this gene family has not been explored in *Ipomoea* species and sweet potato. Methods: A total of 19 *SGR* family genes (*SGRs*) were identified using Basic Local Alignment Search Tool (BLAST) methods. The proteins’ physiological properties, evolutionary and phylogenetic relationships, conserved domain and motifs, gene structures, collinearity, and promoter *cis*-elements were systematically analyzed. Moreover, expression patterns and protein interaction network analyses were performed for sweet potato. Results: In this study, we identified 19 *SGRs* in six *Ipomoea* species. These *SGRs* were divided into four subgroups according to their phylogenetic relationships. Domian analysis revealed that SGRs had the conserved “stay-green” domain. Gene structure analysis showed that *SGRs* had similar structures. The collinearity analysis revealed that the *SGRs* originated from two genes, with one gene undergoing duplication during evolution history; moreover, the *SGRs* experienced rearrangement throughout the evolutionary process in the *Ipomoea* species. *Cis*-elements related to pigment biosynthesis and hormone and stress responses were found. In addition, expression pattern analysis showed that *IbSGRs*, especially *IbSGR1*, *IbSGR2*, and *IbSGR3*, might play an important role in pigment accumulation. The *SGRs* could also respond to stress responses (i.e., cold, drought, and salt) and take part in hormone crosstalk (i.e., abscisic acid (ABA), methyl jasmonate (MeJA), salicylic acid (SA)). Conclusions: Taken together, the findings of this study provide new insights for further understanding the functions of *SGRs* and candidate genes for pigment accumulation and stress tolerance in sweet potatoes.

## 1. Introduction

Chlorophyll is one of the most vital pigments on earth and a key component of photosynthesis [1]. Chlorophyll metabolism is a complex and coordinated process completed by a series of proteins that directly affect the life activities of plants, such as leaf senescence and fruit ripening [2,3]. However, excess chlorophyll generates reactive oxygen species (ROS) that are harmful to plants [3]. Thus, chlorophyll metabolic processes must be finely controlled. Stay-green proteins (SGRs) play a central role in the regulation of plant chlorophyll degradation and senescence, which are relatively conserved in higher plants [4,5]. The SGR superfamily is divided into two subfamilies, including the SGR subfamily and the SGR-like subfamily (SGRL) [6]. So far, there has been genome-wide identification of some of the *SGR* family genes (*SGRs*) in different plant species. For example, two, three, and three *SGRs* were identified in rice [7], *Arabidopsis* [8], and tomato [9], respectively. *CsSGR1*, *CsSGR2*, and *CsSGRL* were identified in tea [10]. Four candidate *SGRs* were identified in melon (*Cucumis melo* L.) [11]. Five *SGRs* were found in each of the genomes of the kiwifruit species [*Actinidia chinensis* (Ac) and *Actinidia eriantha* (Ae)] [6]. However, *SGRs* have not been identified in sweet potato or other *Ipomoea* species. The overexpression of the *SGR* gene causes reductions in chlorophyll contents and cell death in the model species *Arabidopsis* [12] and rice [4,13,14]. In rice, the overexpression of *OsSGRL* reduces the levels of chlorophyll and accelerates its degradation in dark-induced senescence leaves [7]. In fall fescue, the *SGR* family gene *FaNYE1* might play a significant regulatory role in chlorophyll degradation during senescence [15]. *MsSGR*-RNAi transgenic plants enhanced the forage quality of *Alfalfa* [16]. In addition to the leaves, chlorophyll also acts in other organs. In *Arabidopsis*, *SGR*-mediated chlorophyll degradation is critical for detoxification during seed maturation [17]. Additionally, *SGRs* were observed to enhance chlorophyll breakdown in the ripening processes of both tomato and pepper [18,19].

According to previous reports, *SGRs* usually act as regulators in carotenoid accumulation. For example, SlSGR1 directly interacts with a key carotenoid biosynthesis enzyme protein SlPSY1 to regulate lycopene and β-carotene accumulation in tomato [20]. In addition, *SGRs* also play pivotal roles in stress response. The natural mutation of *OsSGR* enhanced resistance to rice sheath blight [21]. The loss of the expression of *CsSGR* (*Cucumis sativus*) improved disease resistance [22]. The overexpression of the *CfSGR1* and *CfSGR2* genes enhanced the stress tolerance of transgenic *Arabidopsis* [23]. The plant hormone abscisic acid (ABA) plays a critical role in leaf senescence and chlorophyll degradation [24]. In *Arabidopsis*, ABA-responsive-element (ABRE) binding transcription factors (TFs), ABF2, ABF3, ABF4, and ABSCISIC ACID INSENSITIVE 3 (ABI3) could directly bind the promoter of *SGR1* and activate its expression [24,25]. In tomatoes, SlABI5 and SlABI5-LIKE could bind to the *SlSGRL* promoter and activate its expression in ABA-induced chlorophyll degradation [26].

*Ipomoea* is one of the largest genera in the Convolvulaceae family. Among them, sweet potato (*Ipomoea batatas* (L.) Lam. [2n = B_1_B_1_B_2_B_2_B_2_B_2_ = 6x = 90]) is an important high-yield, nutritious, hexaploidy storage root crop worldwide [27,28]. It can provide carbohydrates, proteins, dietary fiber, vitamins, and other bioactive compounds, including carotenoids and anthocyanins, and has therefore been considered a vital crop for food and nutrition security [29,30,31,32]. The color of sweet potato flesh ranges from white to cream, yellow, orange, pink, and even purple [33]. Orange-fleshed cultivars, in particular, contain high levels of β-carotene, which could be used to combat vitamin A deficiency, and purple-fleshed cultivars are rich in anthocyanin, which has some beneficial properties such as anti-oxidant, anti-inflammatory, anti-tumor, and anti-mutagenic effects for preventing diabetes, cardiovascular disease, colon cancer, and other diseases [34,35,36,37]. Chlorophyll and carotenoid are two main photosynthetic pigments in plants that play various roles in plant growth and development [38]. Carotenoid accumulation is usually accompanied by the process of chlorophyll degradation [39]. Previous studies have shown that *IbSGR1* might participate in carotenoid accumulation in sweet potatoes. The IbNAC29-IbMYB1R1-IbAITR5 module down-regulates the expression of *IbSGR1* to positively regulate carotenoid accumulation [40]. However, the functions and molecular mechanism of *SGRs* are still unclear in sweet potato and other *Ipomoea* species.

In this study, we identified three, three, three, three, three, and four *SGR* genes (*SGRs*) in the six *Ipomoea* species (i.e., *Ipomoea aquatic*, *I. aquatic*; *Ipomoea cairica*, *I. cairica*; *Ipomoea nil*, *I. nil*; *Ipomoea triloba*, *I. triloba*; *Ipomoea trifida*, *I. trifida* and *Ipomoea batatas*, *I. batatas*), respectively. We identified and systematically analyzed the protein’s physiological properties, evolutionary and phylogenetic relationships, conserved domains and motifs, gene structure, collinearity, and promoter *cis*-elements in six *Ipomoea* species. Their expression patterns associated with carotenoid and anthocyanin contents and stress responses were analyzed using RNA-seq data in sweet potato. This study interpreted the evolutionary relationships of *SGRs* and potential functions of the six *Ipomoea* species, which was helpful to provide valuable insights for further understanding the functions of *SGRs* and candidate genes for enhancing quality traits and improving stress tolerance in sweet potato.

## 2. Materials and Methods

### 2.1. Identification of SGRs

The genome sequences of *I. aquatic*, *I. cairica*, and *I. nil* were obtained from the National Genomics Data Center (NGDC) (https://ngdc.cncb.ac.cn/gwh/Assembly/986/show, accessed on 1 August 2024), the Zenodo repository (https://zenodo.org/records/6792002#.Y90Mb3ZBy4Q, accessed on 1 August 2024), and the Shigen database (http://viewer.shigen.info/asagao/index.php, accessed on 1 August 2024), respectively. In addition, all genome sequences of *I. triloba*, *I. trifida*, and *I. batatas* were downloaded from the *Ipomoea* Genome Hub (www.sweetpotao.com, accessed on 1 August 2024). Basic Local Alignment Search Tool (BLAST) methods were used to identify the *SGRs*. The amino acid sequences of *SGRs* were identified by a previous study of *Arabidopsis thaliana (A. thaliana*) as queries (BLASTP, E value ≤ 1 × 10^−5^). Furthermore, all putative SGRs were checked using the CD-Search in National Center for Biotechnology Information database.

### 2.2. Property Prediction of SGRs

The protein characteristics (i.e., Number of Amino Acids, Molecular Weight, Theoretical pI, Instability Index, Aliphatic Index, and Grand Average of Hydropathicity) were calculated busing the ExPASy tool (https://web.expasy.org/protparam/, accessed on 28 August 2024). The subcellular localization was conducted using the DeepLoc 2.1 server (https://services.healthtech.dtu.dk/services/DeepLoc-2.1/, accessed on 28 August 2024). The secondary of the SGRs were predicted using NetSurfP-3.0 [41].

### 2.3. Phylogenetic Analysis of SGRs

The phylogenetic analysis of *SGRs* was performed using MAFFT with the default parameters in the six *Ipomoea* species [42]. Subsequently, the software TrimAl (version 1.5.0) was used to align the trimming of the sequences [43]. Then, the phylogenetic tree was created using the IQ-TREE 2 with the bootstrap (1000 replicates) and best model being performed automatically [44]. The visualization of the phylogenetic trees was finished using the Interactive Tree of Life (ITOL) platform (https://itol.embl.de/index.shtml, accessed on 15 September 2024).

### 2.4. Domain Identification, Conserved Motif Analysis, Gene Structure Analysis of SGRs

All SGR proteins’ conserved domains were identified using the CD-Search tool (https://www.ncbi.nlm.nih.gov/Structure/cdd/wrpsb.cgi, accessed on 15 August 2024). The conserved motif of SGR proteins was predicted using Multiple Em for Motif Elicitation (MEME, https://meme-suite.org/meme/tools/meme, accessed on 30 September 2024). The gene structures were visualized by the GSDC (https://gsds.gao-lab.org/, accessed on 30 September 2024).

### 2.5. Collinearity Analysis and Classification of Gene Duplication

The BLASTP results were used for the analysis by MCScanX, which conducted the collinearity blocks across the entire genome [45]. The collinearity pairs were extracted and the collinearity map was generated using the CIRCOS software (version 0.69-9) [46]. The duplicate_gene_classifier script of MCScanX was used to analyze the classification of gene duplication.

### 2.6. Cis-Acting Element Analysis of the Promoter of SGRs

The sequence 2000 bp upstream of the promoter regions of *SGRs* was extracted using the TBtools software (version 2.154) [47]. PlantCARE tool (http://bioinformatics.psb.ugent.be/webtools/plantcare/html/, accessed on 15 October 2024) was employed to predict the *cis*-elements of the promoter of *SGRs*. The visualization of the *cis*-element was conducted using the Python package Seaborn (version 0.13.1).

### 2.7. Transcriptome Analysis and Construction of Protein Interaction Network

We downloaded the publicly available RNA sequencing (RNA-seq)-based expression data under the BioProject accession numbers PRJNA881010, PRJNA881014, PRJNA881013, PRJNA881012, PRJNA642259, and PRJNA511028 from the National Genomics Data [48,49,50]. PRJNA881010, PRJNA881014, PRJNA881013, and PRJNA881012 are transcriptome datasets for four sweet potato varieties with different flesh colors: 1143-1 (white flesh), HS (orange flesh), DZ88 (purple flesh), and DZ54 (purple flesh). PRJNA642259 is the transcriptome dataset for the *I. batatas* varieties XS-18 (white flesh) and XZS-3 (purple flesh). PRJNA511028 is the transcriptome dataset for the sweet potato variety Xu18’s tissues leaves, stems, and fibrous roots, treated under hormone treatments (ABA, MeJA, SA) and abiotic stress treatments (cold, drought, and salt). Firstly, the adapt and low-quality reads were removed by Fastp [51]. Secondly, the obtained reads were mapped to the sweet potato reference (downloaded from the *Ipomoea* Genome Hub genome) using STAR software (version 2.7.10b) [52]. Finally, the Featurecount was used to obtain the abundance matrix of the exons [53]. We used an in-house Python script to calculate the Transcripts Per Kilobase of the exon model per Million mapped reads (TPM) values for the *SGRs*. The multiple tissue expression patterns of sweet potato were normalized using log_2_ (TPM+1). Furthermore, a heatmap was generated using the Seaborn package in Python.

Based on the IbSGR protein sequences, the interaction networks of the IbSGRs were constructed using the STRING database (https://string-db.org/, accessed on 20 November 2024) and the AlphaFold 3 database (https://alphafoldserver.com/, accessed on 10 December 2024). The interaction networks were visualized using PyMOL software (version 3.1.3).

## 3. Results

### 3.1. Identification and Characterization of SGRs in the Six Ipomoea Species

**Figure 1:**
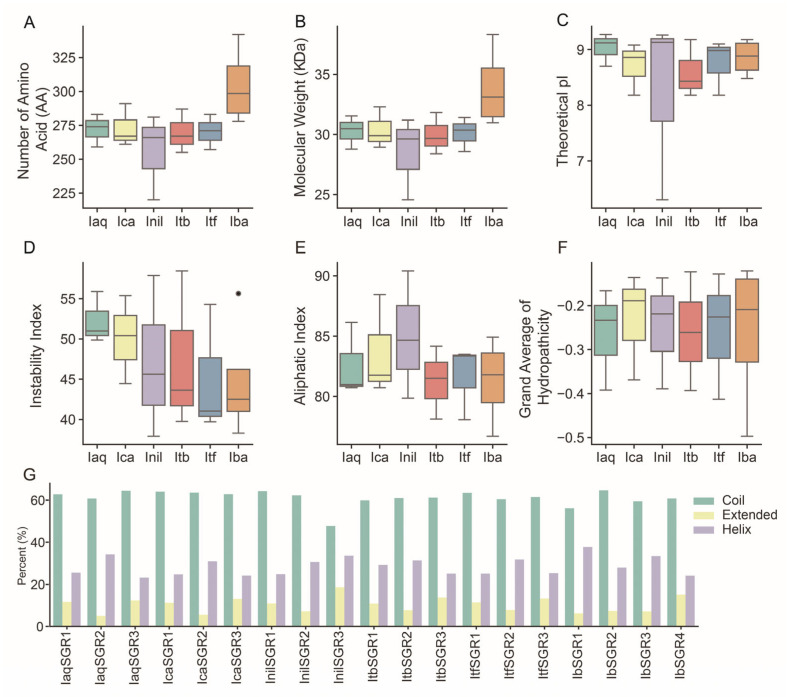
Analysis of physical and chemical characteristics of SGR proteins in six *Ipomoea* species. (**A**) Number of amino acids of SGR proteins. (**B**) Molecular weight of SGR proteins. (**C**) Theoretical pI of SGR proteins. (**D**) Instability index of SGR proteins; the black dots represent excessively high values of the instability index. (**E**) Aliphatic index of SGR proteins. (**F**) Grand average of hydropathicity of SGR proteins. (**G**) Predicted secondary structures of SGR proteins. *Ipomoea aquatica*, Iaq; *Ipomoea cairica*, Ica; *Ipomoea nil*, Inil; *Ipomoea triloba*, Itb; *Ipomoea trifida*, Itf; *Ipomoea batatas*, Iba.

In this study, a total of 19 *SGR* members were extensively identified using Basic Local Alignment Search Tool (BLAST) searches in *I. aquatica* (*IaqSGRs*), *I. cairica* (*IcaSGRs*), *I. nil* (*InilSGRs*), *I. triloba* (*ItbSGRs*), *I. trifida* (*ItfSGRs*) and *I. batatas* (*IbSGRs*) (Table S1). The protein physiological properties of SGRs were analyzed using the sequences in the six *Ipomoea* species. The CDS length of the *SGRs* varied from 663 bp (*InilSGR3*) to 1029 bp (*IbSGR1*) (Table S1). The length of putative proteins ranged from 220 aa (InilSGR3) to 342 aa (IbSGR1), with a molecular weight (MW) of 24.56 kDa (InilSGR3) to 38.32 kDa (IbSGR1) and a theoretical isoelectric point (pI) of 6.3 (InilSGR3) to 9.27 (IaqSGR2) (Figure 1A–C, Table S1). Except for InilSGR3, IbSGR4, ItfSGR2, and ItbSGR3, the instability index of other proteins was more than 40 (Figure 1D, Table S1). The aliphatic index of SGRs ranged from 76.7 (IbSGR1) to 90.41 (InilSGR3) (Figure 1E, Table S1). The grand average of hydropathicity (GRAVY) values of SGRs varied from −0.497 to −0.121, indicating that they are hydrophilic proteins (Figure 1F, Table S1). Subcellular localization prediction showed that most of the SGRs were located in plastids except for ItbSGR1 and InilSGR1 in the cytoplasm, IbSGR3 in the endoplasmic reticulum, and InilSGR3 in the nucleus (Table S1). A secondary structure analysis of SGRs showed that the coil structure was dominant in all SGRs and the ratio of the coil structure was between 47.73% (InilSGR3) and 64.69% (IbSGR2) (Figure 1G).

### 3.2. Evolutionary and Phylogenetic Relationship Analysis of SGRs in the Six Ipomoea Species

**Figure 2:**
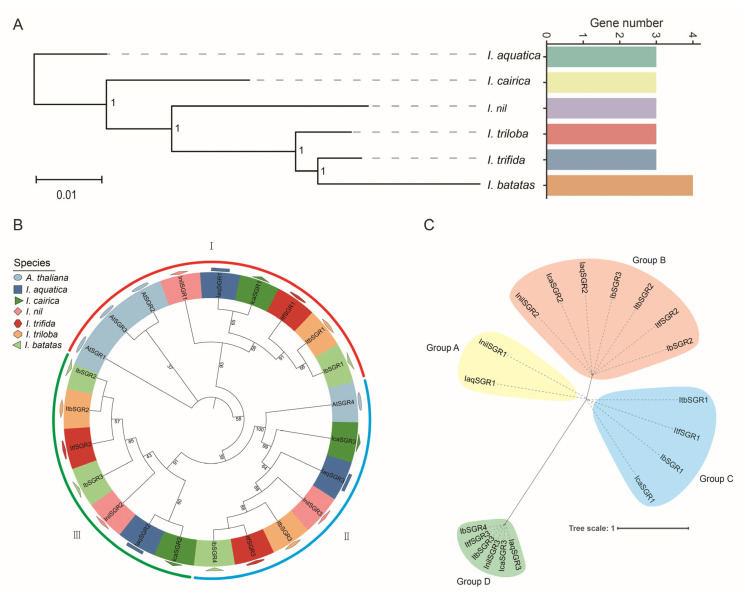
Evolutionary and phylogenetic analysis of the *SGRs* in six *Ipomoea* species. (**A**) Evolutionary and phylogenetic analysis of the *SGRs* in *I. aquatica*, *I. cairica*, *I. nil*, *I. triloba*, *I. trifida*, and *I. batatas*. The phylogenetic tree of the six *Ipomoea* species is shown on the left, and the number of *SGR* members of different species is shown on the right. Values at the nodes indicate bootstrap support (1 = 100%). (**B**) Phylogenetic tree of *SGRs* in *Arabidopsis thaliana* (*A. thaliana*) and the six *Ipomoea* species. The *SGRs* were classified into three groups (I, II, and III) based on their similarity. Different species were marked in different colors and shapes. (**C**) Phylogenetic tree of *SGRs* in six *Ipomoea* species. 19 *SGRs* were divided into four groups (groups A, B, C, and D filled with yellow, pink, blue, and green, respectively).

Constructing the species evolutionary tree and counting the number of *SGR* members in different species showed that the gene number of *SGRs* was increased during the evolutionary history (Figure 2A). In order to characterize the evolutionary relationships of *SGRs* in *Arabidopsis thaliana* (*A. thaliana*) and the six *Ipomoea* species, we constructed a phylogenetic tree for 23 SGRs of these seven species (i.e., four in *A. thaliana*, three in *I. aquatica*, three *in I. cairica*, three in *I. nil*, three in *I. triloba*, three in *I. trifida*, and four in *I. batatas*). These SGRs were divided into three subgroups together with their orthologous SGRs from *Arabidopsis*, including Group I (AtSGR1/2/3, InilSGR1, IaqSGR1, lcaSGR1, ItfSGR1, ItbSGR1, and IbSGR1), Group II (AtSGR4, lcaSGR3, IaqSGR3, InilSGR3, ItbSGR3, ItfSGR3, and IbSGR4) and Group III (lcaSGR2, IaqSGR2, InilSGR2, IbSGR3, ItfSGR2, ItbSGR2, and IbSGR2) (Figure 2B). In addition, we constructed a phylogenetic tree with evolutionary distances of the six *Ipomoea* species, and all *SGRs* were divided into four groups with uneven distribution in each group. Interestingly, both IbSGR2 and IbSGR3 were in Group B, revealing that gene expansion occurred within this species during the evolution process (Figure 2C).

### 3.3. Conserved Domain and Motif Analysis of SGRs in the Six Ipomoea Species

**Figure 3:**
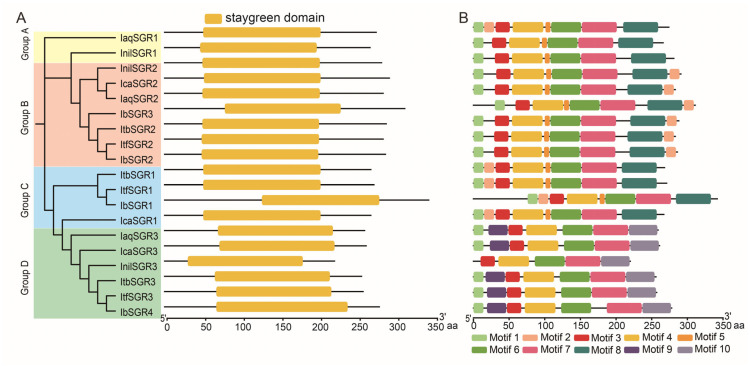
Conserved domain and motif analysis of SGRs in six *Ipomoea* species. (**A**) Phylogenetic tree showing SGRs divided into three subgroups on the left. These genes are divided into four groups according to the evolutionary tree (groups A, B, C, and D, filled with yellow, pink, blue, and green, respectively). Stay-green domain of SGRs was marked orange. (**B**) Ten conserved motifs were identified in SGRs. Motifs were shown in different colors.

We analyzed the domain of SGR proteins, and the results showed that all SGRs contained the stay-green domain (Figure 3A). Moreover, we analyzed the sequence motifs of SGR proteins through the MEME website and identified the ten conserved motifs (Figure 3B). The majority of the SGRs contained most of the motifs except for motif 9 and motif 10, including IaqSGR1/2, IcaSGR1/2, ItbSGR2, ItfSGR2, and IbSGR1/2/3. In addition, IaqSGR3, IcaSGR3, ItbSGR3, ItfSGR3, and IbSGR4 contained seven motifs except for 2, 5, and 8 (Figure 3B). In addition, InilSGR1/2, ItbSGR1, and ItfSGR1 had seven motifs except for 2, 9, and 10. InilSGR3 contained the least number of motifs, only including motifs 3, 4, 6, 7, and 10 (Figure 3B).

### 3.4. Gene Structure Analysis of SGRs in the Six Ipomoea Species

**Figure 4:**
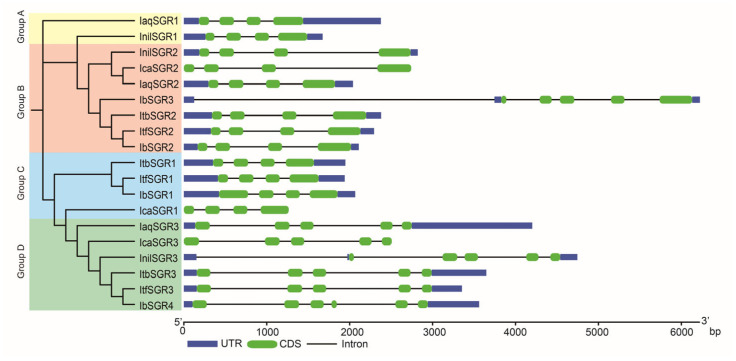
Exon–intron structures of *SGRs* in six *Ipomoea* species. The phylogenetic tree shows *SGRs* divided into four subgroups on the left. These genes are divided into four groups according to the evolutionary tree (groups A, B, C, and D, filled with yellow, pink, blue, and green, respectively). The purple boxes, green boxes, and black lines represent the UTRs, CDS, and introns, respectively.

To better understand the structural features of *SGR* genes, gene structures were analyzed based on their evolutionary relationships. Overall, the gene structure of *SGRs* showed small variations. The number of CDS and introns is 4, 5, or 6 and 3, 4, or 5, respectively (Figure 4). Here, 10 of 19 members of *SGRs* contained two UTRs, four CDS, and three introns in the six *Ipomoea* species, including all members of group A (*IaqSGR1* and *InilSGR1*), most of the members of group B (except for *IcaSGR2* and *IbSGR3*) and *ItbSGR1*, *ItfSGR1*, and *IbSGR1* in group C (Figure 4). Most of the members of group D contained five CDSs except for *IbSGR4*, which had six CDSs (Figure 4). Interestingly, no UTR of the gene structure of *SGRs* was detected in *I. cairica*. In general, most of the *SGRs* in the same groups showed similar gene structures, which supports their close evolutionary relationship (Figure 4). *IbSGR1/2*, *IbSGR3*, and *IbSGR4* contained four, five, and six CDSs, respectively. These results indicate that the *SGR* gene family might become more complex in the evolution process.

### 3.5. Collinearity Analysis of SGRs in the Genomes of the Six Ipomoea Species

**Figure 5:**
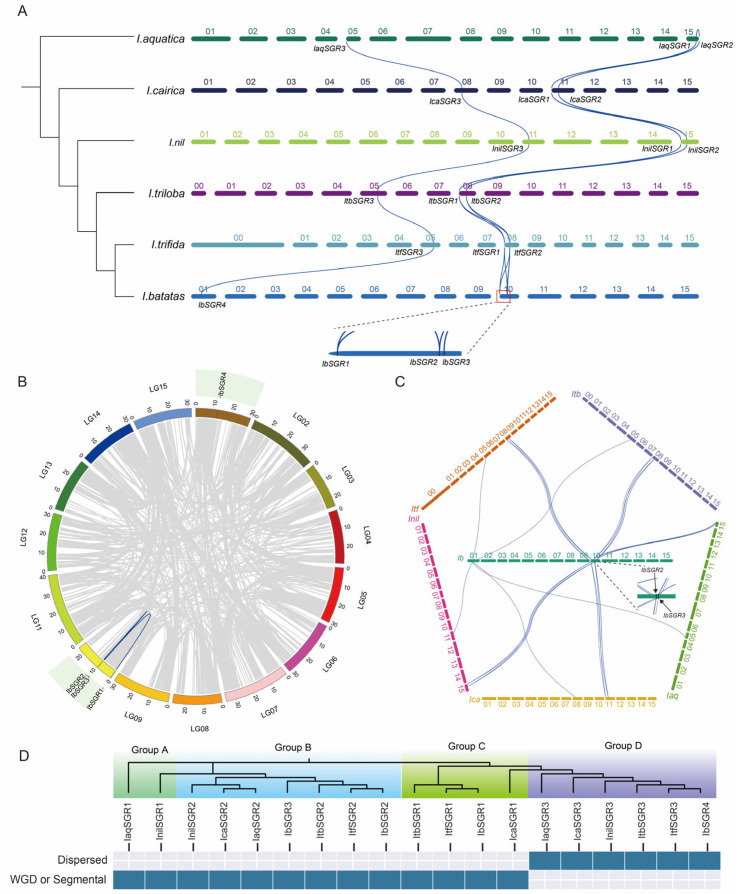
Collinearity analyses of *SGRs* in six *Ipomoea* species. (**A**) Collinearity analysis of the six *Ipomoea* species *SGRs*. The phylogenetic tree of the six *Ipomoea* species is shown on the left. Chromosomes of *I. aquatica*, *I. cairica*, *I. nil*, *I. triloba*, *I. trifida*, and *I. batatas* are shown in different colors. Blue curves denote the collinearity relationships of *SGRs* in six *Ipomoea* species. (**B**) Chromosomal localization and distribution of *SGRs* in *I. batatas*. The relative chromosomal localization of each *SGR* gene is marked on the short black lines. Blue curves denote the collinearity relationships of *IbSGRs*. (**C**) Collinearity analysis between *I. batatas* and the other five *Ipomoea* species. Blue curves denote the collinearity relationships. (**D**) Duplication modes of *SGRs* in six *Ipomoea* species.

To further investigate the evolution of *SGR* genes of the *Ipomoea* genus, we conducted interspecies and intraspecies collinearity analyses (Figure 5 and Figure S1). The results revealed that *IaqSGR3*, *IcaSGR3*, *InilSGR3*, *ItbSGR3*, *ItfSGR3*, and *IbSGR4* exhibited collinearity, indicating that they originated from a common ancestral gene (Figure 5A). Additionally, *IaqSGR1* and *IcaSGR1*, as well as *IcaSGR2*, exhibited collinearity with *IaqSGR2* and *IcaSGR1*, suggesting a shared evolutionary origin. Furthermore, *IcaSGR1*, *IcaSGR2*, *InilSGR1*, *InilSGR2*, *ItbSGR1*, *ItbSGR2*, *ItfSGR1*, *ItfSGR2*, *IbSGR1*, *IbSGR2*, and *IbSGR3* also exhibited collinearity. Moreover, the analysis of collinearity within *I. aquatica* showed that *IaqSGR1* and *IaqSGR2* exhibit synteny, suggesting that *IaqSGR1*, *IaqSGR2*, *IcaSGR1*, *IcaSGR2*, *InilSGR1*, *InilSGR2*, *ItbSGR1*, *ItbSGR2*, *ItfSGR1*, *ItfSGR2*, *IbSGR1*, *IbSGR2*, and *IbSGR3* originated from a common ancestral gene (Figure 5A and Figure S1A). In addition, we performed synteny analysis within the *I. batatas* species and found that *IbSGR1* and *IbSGR3* exhibited collinearity, as did *IbSGR3* and *IbSGR2* (Figure 5B). However, no collinearity was observed between *IbSGR1* and *IbSGR2*, indicating more complex chromosomal variations in the *IbSGR1* and *IbSGR2* regions within *I. batatas* (Figure 5B). Furthermore, we conducted a collinearity analysis between the *SGR* genes of *I. batatas* and the other five species, revealing that the *SGR* genes of *I. batatas* are highly conserved within the *Ipomoea* genus (Figure 5C). The Duplicate_gene_classifier tool in MCScanX was employed to ascertain the duplication modes of *SGR* genes in *Ipomoea* species. The primary duplication mode of *SGR* genes in *Ipomoea* species was identified as WGD or Segmental, and dispersed duplication (Figure 5D). To determine the within-species collinearity relationships of the other five *Ipomoea* species, we conducted a collinearity analysis. The results showed that within the *SGR* genes of these five species, only one pair of *SGR* genes exhibited collinearity in each species, while two pairs of genes in sweet potato displayed collinearity. This difference may be due to the duplication of *SGR* genes within sweet potato (Figure S1).

### 3.6. Cis-Element Analysis in the Promoters of SGRs in the Six Ipomoea Species

**Figure 6:**
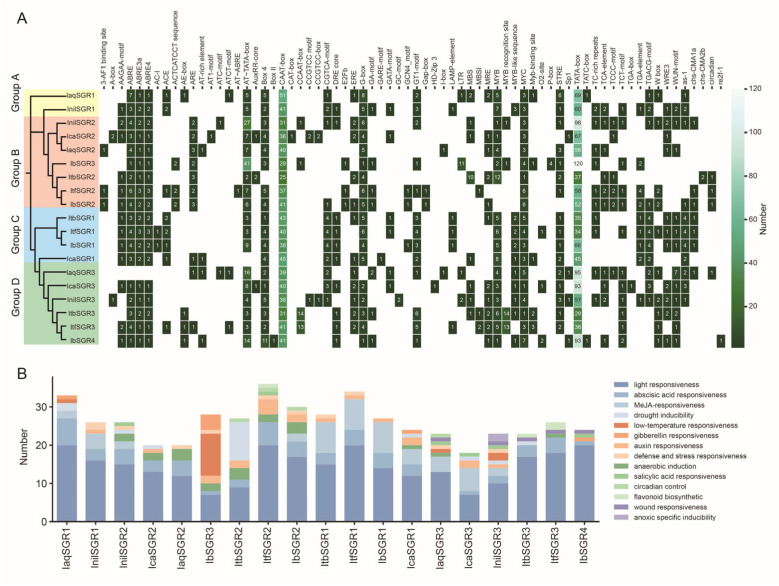
*Cis*-element analysis in the promoters of *SGRs* in six *Ipomoea* species. (**A**) Heatmap of *cis*-elements in the promoters of *SGRs.* The degree of green colors represents the number of cis-elements in the promoters of *SGRs*. (**B**) Summary and classification of the functions of *cis*-elements. Different colors represent different functions.

The *cis*-acting regulatory elements were analyzed using 2000 bp promoter sequences upstream of the coding region to explore the potential functions of *SGRs* in the six *Ipomoea* species. The results showed that all of the *SGR* promoters had a large number of core/binding elements, including CAAT-box and TATA-box, and the light-responsive elements existed in all of the *SGR* promoters (Figure 6A). Some growth, development, and pigment biosynthesis elements were detected in the promoters of *SGRs*. For example, the O2-site (zein metabolism regulatory element) was found in the promoters of *IcaSGR3*, *IbSGR4*, and *ItfSGR1;* the MBSI motif (MYB binding site involved in flavonoid biosynthetic gene regulation) was found in the promoters of *ItfSGR2/3* and *ItbSGR3* (Figure 6A). In addition, most of the *SGRs* contained abundant hormone-responsive elements such as abscisic acid-responsive elements ABRE, ABRE3a, and ABRE4; MeJA-responsive elements CGTCA-motif and TGACG-motif; gibberellin-responsive elements P-box and GARE-motif; auxin-responsive elements TGA-element and AuxRR-core; and the salicylic-acid-responsive element TCA-element. In addition, the biotic/abiotic elements including TC-rich repeats (defense- and stress-responsive elements), LTR (low-temperature responsive elements), ARE (aerobic induction-responsive elements), and MBS (MYB binding site involved in drought inducibility) were present in the promoter of *SGRs*. Furthermore, all promoters of *IbSGRs* contained ABA-responsive elements (Figure 6A). We detected the W-box in the promoter of *IaqSGR1/3*, *InilSGR2/3*, *ItbSGR3*, *ItfSGR1/2/3*, and *IbSGR2/4*, which might be bounded by WRKY TFs. Some of the promoters of *SGRs* (i.e., *laqSGR1*, *lcaSGR1*, *ltbSGR3*, *ltfSGR3*, and *IbSGR3*) had the MYB-binding site, which might be regulated by MYB TFs (Figure 6A). We then classified the functions of the elements and found that the *SGR* genes contain the highest number of light-responsive elements, followed by ABA-responsive elements. Some genes, such as *IbSGR1*, contain a relatively simple set of elements, indicating that this gene has simpler transcriptional regulation compared to other SGR genes (Figure 6B). These results suggested that *SGRs* might be involved in the regulation of plant growth and development and pigment biosynthesis, and may participate in phytohormone and abiotic/biotic stress adaption signaling pathways.

### 3.7. Expression Patterns of IbSGRs

#### 3.7.1. Expression Patterns of *IbSGRs* in Different Flesh-Colored Sweet Potato Varieties

**Figure 7:**
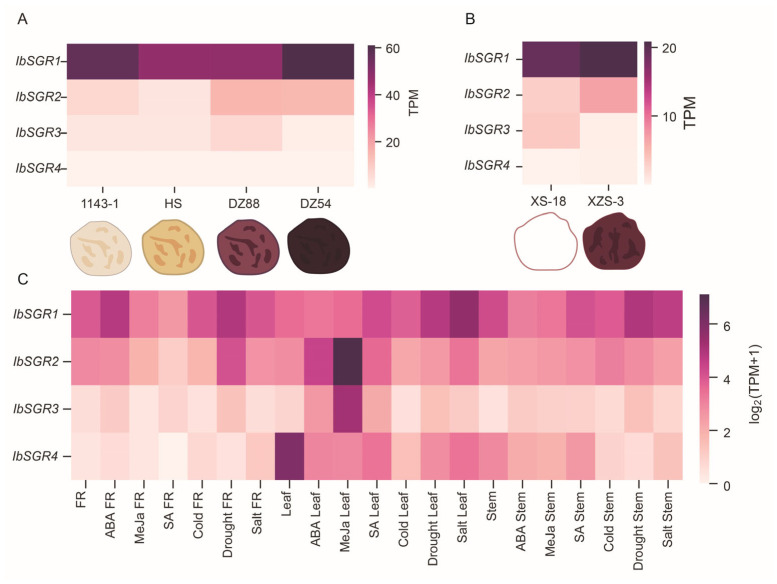
Gene expression patterns of *IbSGRs*. (**A**) Gene expression patterns of *IbSGRs* in four sweet potato materials: 1143-1 (white flesh), HS (orange flesh), DZ88 (purple flesh), and DZ54 (purple flesh). (**B**) Gene expression patterns of *IbSGRs* in different sweet potato materials: XS-18 (white flesh), XZS-3 (purple flesh). (**C**) Gene expression patterns of *IbSGRs* in response to different phytohormones and stresses (i.e., ABA, MeJA, SA, cold, drought, and salt) in the leaves, stems, and fibrous roots, respectively.

The RNA-seq data (PRJNA881010, PRJNA881014, PRJNA881013, and PRJNA881012) of sweet potato with different flesh colors, including the four sweet potato varieties 1143-1 (white flesh), HS (orange flesh), DZ88 (purple flesh), and DZ54 (purple flesh). The RNA-seq data (PRJNA642259) of XS-18 (white flesh) and XZS-3 (purple flesh) were analyzed to investigate whether *IbSGRs* had an effect on pigment biosynthesis. We discovered that the expression level of *IbSGR1* and *IbSGR2* in white-fleshed 1143-1 is lower than that of orange-fleshed HS, which might suggest that *IbSGR1* and *IbSGR2* play negative roles in carotenoid biosynthesis (Figure 7A). In addition, for the biosynthesis of anthocyanin, the expression level of *IbSGR1*, *IbSGR2*, and *IbSGR3* was negatively correlated with anthocyanin accumulation (Figure 7A,B). The expression of *IbSGR4* was relatively stable in sweet potato varieties with different-colored flesh (Figure 7A,B). Taken together, these results show that *IbSGRs* possess different expression patterns and play vital roles in the carotenoid and anthocyanin biosynthesis of sweet potato.

#### 3.7.2. Expression Patterns of *IbSGRs* in Different Hormones and Stress Responses

We analyzed the previously published RNA-seq data of three tissues, including FR (fibrous root), leaf, and stem, for different hormones and stress responses (i.e., ABA, MeJA, SA, cold, drought, and salt) to discover the potential function of the *IbSGRs*. The *IbSGRs* were expressed in the FRs, leaves, and stems. The *IbSGRs* in different tissues were induced by various treatments to different degrees (Figure 7C). Above all, these results indicated that some *IbSGRs* are involved in the response to hormones and stresses in sweet potato.

### 3.8. Protein Interaction Network of IbSGRs in Sweet Potato

**Figure 8:**
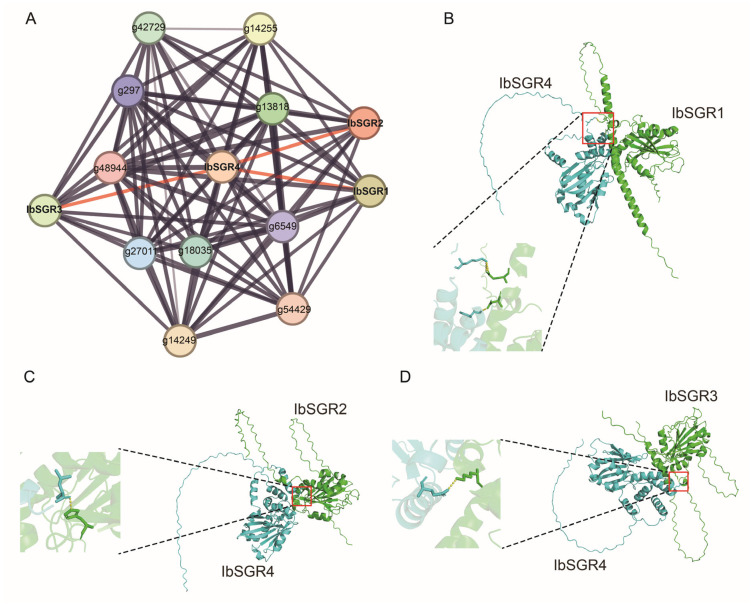
The interaction protein networks of *IbSGRs*. (**A**) The interaction protein networks of IbSGRs predicted by the STRING database. Network nodes represent proteins, and lines represent protein–protein associations. The gray lines represent physical interactions and the thickness of the lines represent the strong interactions of *IbSGRs* with each other. The red boxes represent the interactions between IbSGR4 and other IbSGRs proteins. (**B**–**D**) The interactions between IbSGR4 and IbSGR1, IbSGR4 and IbSGR2, and IbSGR4 and IbSGR3 predicted by alphafold3. The IbSGR4 protein is marked in cyan, and the other IbSGR proteins are marked in green. The interaction sites are marked by red boxes. In the enlarged view of the interaction site, the yellow dashed lines represent the interaction sites.

To investigate the potential regulatory network of IbSGRs, we constructed an IbSGR interaction network using the STRING database (Figure 8A). Consistent with the function of SGR proteins, IbSGRs usually interact with proteins in chloroplasts. For example, IbSGRs can interact with chlorophyll synthase (g18035 and g27011), chlorophyllase 2 (g13818), and protochlorophyllide reductase (g297 and g54429) (Figure 8). Interestingly, IbSGR4 can interact with IbSGR1, IbSGR2, and IbSGR3, which might mean that IbSGR4 synergistically works with other IbSGR proteins (Figure 8A). Then, we further investigated the interaction between IbSGR4 and the other three IbSGR proteins using AlphaFold 3, which indicated that IbSGR4 can interact with the other three IbSGR proteins (Figure 8B–D).

## 4. Discussion

### 4.1. Evolution of SGRs in the Six Ipomoea Species

So far, *SGR* genes have been identified in some plant species using comparative genome analysis methods [7,8,9,10,11,12]. Two *SGRs* were found in rice [7]. *Arabidopsis*, tomato, and tea all have three *SGRs* [8,9,10]. Melon has four *SGRs*, and kiwifruit has five *SGRs* [11,12]. In this study, three, three, three, three, three, and four *SGRs* were identified in *I. aquatic*, *I. cairica*, *I. nil*, *I. triloba*, *I. trifida*, and *I. batatas*, respectively. The results suggested that the number of *SGRs* was similar among different species, which indicated that *SGR* gene members are relatively conserved during the evolutionary process. In addition, the number of *SGRs* in sweet potato was one more than in other species. This indicated that the *SGR* gene family expanded during the evolutionary history of the six *Ipomoea* species. According to the phylogenetic tree analysis, the *SGR* gene family has undergone expansion during its evolutionary history and it occurs in group B (Figure 2). In order to further explore the origin and evolution of the *SGR* gene family, collinearity analysis was performed and revealed that the *SGR* gene family originated from two genes, with one gene undergoing duplication. WGD or Segmental duplication events play a dominant role in expanding the *SGR* gene family (Figure 5).

In plants, the domain is evolutionarily conserved units of proteins, which are widely used to classify protein sequences and predict protein functions [54]. Furthermore, the domain structure of a protein is usually associated with its subcellular localization, interactions, and other functions of the protein [55,56]. Previous studies have suggested that single amino acid substitutions within this domain could obtain the mutant of *SGRs* [4,14,57]. In our study, all SGR proteins had the conserved stay-green domain and similar properties, which might mean that they have similar functions (Figure 1 and Figure 3). These results are consistent with previous reports, which found that the functions of SGR proteins are highly conserved in different plants [58]. Most of the SGRs are located in plastids, which is consistent with their work mainly in chloroplasts (Table S1). In addition, the different motif structure of IbSGR1/2/3 to that in IbSGR4 demonstrated the relative diversity of protein structures (Figure 3B).

Gene structure analysis is of great significance to the study of gene functions [59]. Based on the gene structure analysis, most of the *SGRs* had the same number of CDSs and introns. For example, all members of groups A, B, and C contained four CDSs and three introns, except *IbSGR3* (Figure 4). These results showed that the gene structure pattern was relatively conservative in the six *Ipomoea* species. However, the three patterns in sweet potato include two UTRs–four CDSs–three introns, two UTRs–five CDSs–five introns, and two UTRs–six CDSs–five introns, which might result in their functional differences (Figure 4). These results indicate that *IbSGRs* went through structural diversification in the process of evolution. Taken together, the findings show that the *SGR* gene family members increased, the conserved domain structure was relatively stable, and the gene structure underwent some changes in the process of evolution.

### 4.2. IbSGRs Are Involved in Carotenoid and Anthocyanin Accumulation in Sweet Potato

*SGRs* are generally reported to participate in plant chlorophyll degradation and senescence, which are closely related to plant growth and development and stress responses [4,5,6,7,8,9,10,11,12,13,14,15,16,17,18,19,20,21,22,23,24]. The normal operation of chlorophyll metabolism is necessary for plants to maintain normal life activities [1,2,3]. *SGR1* was demonstrated as the Mg-dechelatase responsible for catabolizing which was an important part of the chlorophyll breakdown [60]. The main function of *SGRs* is to regulate plant chlorophyll degradation and this has been confirmed in various species such as *Arabidopsis* [17], rice [13], tomato [26], and *Medicago truncatula* [16]. Chlorophylls and carotenoids are essential and beneficial substances for both plant and human health, and there is a certain connection between the synthesis mechanisms of these two pigments. Carotenoid accumulation is usually accompanied by the process of chlorophyll degradation [38,39]. In tomatoes and peppers, *SGRs* are involved in the regulation of color change during fruit ripening [18,19]. Interestingly, some studies have shown that *SGRs* also play a regulatory role in the biosynthesis of carotenoids. SlSGR1 directly interacts with SlPSY1 to play a pivotal regulatory role in color formation and fruit ripening regulation in tomato [20]. As reported by Biswal [61], the levels of both chlorophyll and carotenoid decline during leaf senescence. Hence, it was proposed that *SGRs* may act as a key factor in the regulation of carotenoid metabolism.

Sweet potato is an essential part of food and nutritional security [27,28]. The flesh of sweet potato comes in many colors and is rich in numerous pigments and nutrients [29,30,31,32,33,34,35,36]. Carotenoids and anthocyanins are important nutrients in sweet potato. Previous studies have shown that the downregulation of *IbSGR1* expression increases carotenoid accumulation [40]. Previous research has also indicated that the expression of *SGR* genes is upregulated during leaf senescence and chlorophyll breakdown [4,14]. The analysis of gene expression levels is helpful for better functional prediction. In our study, according to the analysis of RNA-seq, *IbSGR1* and *IbSGR2* played negative roles in carotenoid biosynthesis, and *IbSGR1*, *IbSGR2*, and *IbSGR3* acted as negative regulators in anthocyanin biosynthesis (Figure 7A). The results showed that *SGRs* might play an important role in carotenoid accumulation in sweet potato. The protein–protein interaction network is a key component in understanding protein functions and building protein regulatory networks [62]. The prediction of the protein interaction network showed that SGRs can interact with chlorophyll synthesis (Figure 8).

Most recent studies have found that WRKY TFs play important roles in carotenoid and anthocyanin biosynthesis and usually act by regulating the expression of other genes. W-box acts as a binding site sequence for WRKY TFs [63]. In citrus, *CrWRKY42* regulates chlorophyll degradation and carotenoid biosynthesis [39]. SlWRKY35 can directly activate the expression of the *SlDXS1* gene to enhance carotenoid accumulation in tomato [64]. *OfWRKY3* positively regulates the carotenoid cleavage dioxygenase gene *OfCCD4* in *Osmanthus fragrans* [65]. The overexpression of *MdWRKY75* enhances the accumulation of anthocyanins in apple (*Malus domestica*) [66]. In our study, the transcription factor (TF) binding sites were analyzed on the promoter of *SGRs*. *IbSGR2* and *IbSGR4* had the WRKY TFs binding site W-box in their promoter, which may mean that *IbSGR2* and *IbSGR4* might be bound and regulated by WRKY TFs to affect pigment accumulation (Figure 6). In addition, MYB TFs occupy a dominant position in the regulatory network of anthocyanin biosynthesis [67,68]. For example, in tomato, *SlMYB75* promotes anthocyanin accumulation and enhances volatile aroma production [69]. *MdMYB3* is involved in the regulation of anthocyanin biosynthesis and flower development in apple [70]. BoMYBL2b inhibits anthocyanin accumulation via directly repressing *BoDFR1* gene transcription in kale [71]. PdMYB118 directly interacts with bHLH transcription factor *PdTT8* to regulate wound-induced anthocyanin biosynthesis in poplar [72]. By analyzing the *cis*-acting elements in the promoters of *SGRs*, we found that *IbSGR3* had an MYB-binding site, which may imply that MYB TFs regulate the expression of *IbSGR3* to influence anthocyanin accumulation (Figure 6 and Figure 7). Taken together, these results indicate that *IbSGRs* may be involved in the response to carotenoid and anthocyanin accumulation in sweet potato.

### 4.3. IbSGRs Regulate Hormone and Stress Responses in Sweet Potato

*SGRs* are reported to regulate the response to ABA in plants. In *Arabidopsis*, ABF2, ABF3, ABF4, and ABI3 can all bind to the promoter of *SGR1* to activate its expression [24,25]. *SlSGRL* can be activated by SlABI5 in tomato [26]. However, the regulation mechanism of *SGRs* in response to hormones is largely unexplored. In our research, we found that ABA-, MeJA-, gibberellin-, auxin-, and SA-responsive elements were present in the *SGR* promoters (Figure 6). The promoter of *IbSGR1* and *IbSGR2* had ABA-, MeJA- and auxin-responsive elements, the *IbSGR3* promoter had ABA- and gibberellin-responsive elements, and *IbSGR4* had gibberellin- and SA-responsive elements (Figure 6). The RNA-seq analysis results showed that *IbSGRs* were induced by ABA, MeJA, and SA (Figure 7). These results indicate that *SGRs* may participate in hormone crosstalk in sweet potato.

*SGRs* are the key regulator involved in keeping plants “stay-green”, which allows plants to keep their leaves on the active photosynthetic level under stress conditions [73]. *OsSGR* was a negative regulator in rice sheath blight [21]. *CsSGR* negatively regulates disease resistance in citrus [22]. In *Arabidopsis*, *AtSGR1* and *AtSGRL* act synergistically for rapid chlorophyll degradation before senescence under salt stress [74]. However, there is no research reporting whether *SGRs* play a role in the stress responses of sweet potato and other *Ipomoea* species. In this research, drought inducibility-, low-temperature-, defense- and stress-, anaerobic induction-, wound-, and anoxic-specific-inducibility-responsive elements were present in the promoters of *SGRs* (Figure 6). Furthermore, the promoters of *IbSGR2* had the defense- and stress- and the anaerobic induction responsiveness elements; the promoters of *IbSGR3* contained low-temperature, anaerobic induction, and anaerobic induction responsiveness elements; and the promoters of *IbSGR4* had wound responsiveness elements (Figure 6). Furthermore, *IbSGRs* were induced by cold, drought, and salt treatment based on the RNA-seq. Interestingly, the FRs, leaves, and stems of *IbSGR3* were all downregulated under cold stress, which might mean that *IbSGR3* is a key negative regulator factor at low temperatures (Figure 7C). Taken together, these results suggest that *SGRs* may regulate the response to biotic and abiotic stress.

## 5. Conclusions

In summary, our results demonstrated that a total of 19 SGR members were identified in *I. aquatica*, *I. cairica*, *I. nil*, *I. triloba*, *I. trifida*, and *I. batatas*. There is one more *IbSGR* member than in the other five species. The SGR proteins had the conserved stay-green domain and similar properties. The *SGR* genes of the six *Ipomoea* species originated from two distinct *SGR* ancestral genes. Most of the *SGR* genes had two exons and three introns. The promoters of the *SGRs* had the *cis*-elements of phytohormone and stress responsiveness. We found that some of the promoters of *IbSGRs* had pigment-associated TF (WRKY and MYB) binding factors. *IbSGR2* and *IbSGR4* had a WRKY TFs binding site and *IbSGR3* had an MYB-binding site in their promoters. In addition, the expression of *IbSGR1*, *IbSGR2*, and *IbSGR3* were related to the color of the sweet potato flesh. *IbSGR3* might be a potential candidate gene to negatively regulate low-temperature responses according to our results. This work provides valuable insights into the evolution and functions of *SGRs* and also supplies valuable candidate genes for improving carotenoid and anthocyanin contents and stress tolerance in sweet potatoes.

## Data Availability

The original contributions presented in the study are included in the article/Supplementary Materials, further inquiries can be directed to the corresponding author.

## Supplementary Materials

The following supporting information can be downloaded at https://www.mdpi.com/article/10.3390/genes16030266/s1: Figure S1: Intraspecific collinearity analysis of SGRs in five *Ipomoea* species; Table S1: Characteristics of identified SGRs in six *Ipomoea* species.
