# Supplementary material for: Systematic Analysis of Stay-Green Genes in Six Ipomoea Species Reveals the Evolutionary Dynamics, Carotenoid and Anthocyanin Accumulation, and Stress Responses of Sweet Potato
Source: Genes (Basel). 2025 Feb 24;16(3):266. doi: 10.3390/genes16030266 (PMC11941861; doi:10.3390/genes16030266)
Supplement: Supplementary file 1 [file genes-16-00266-s001.zip › SGR-fingerS1.pdf]

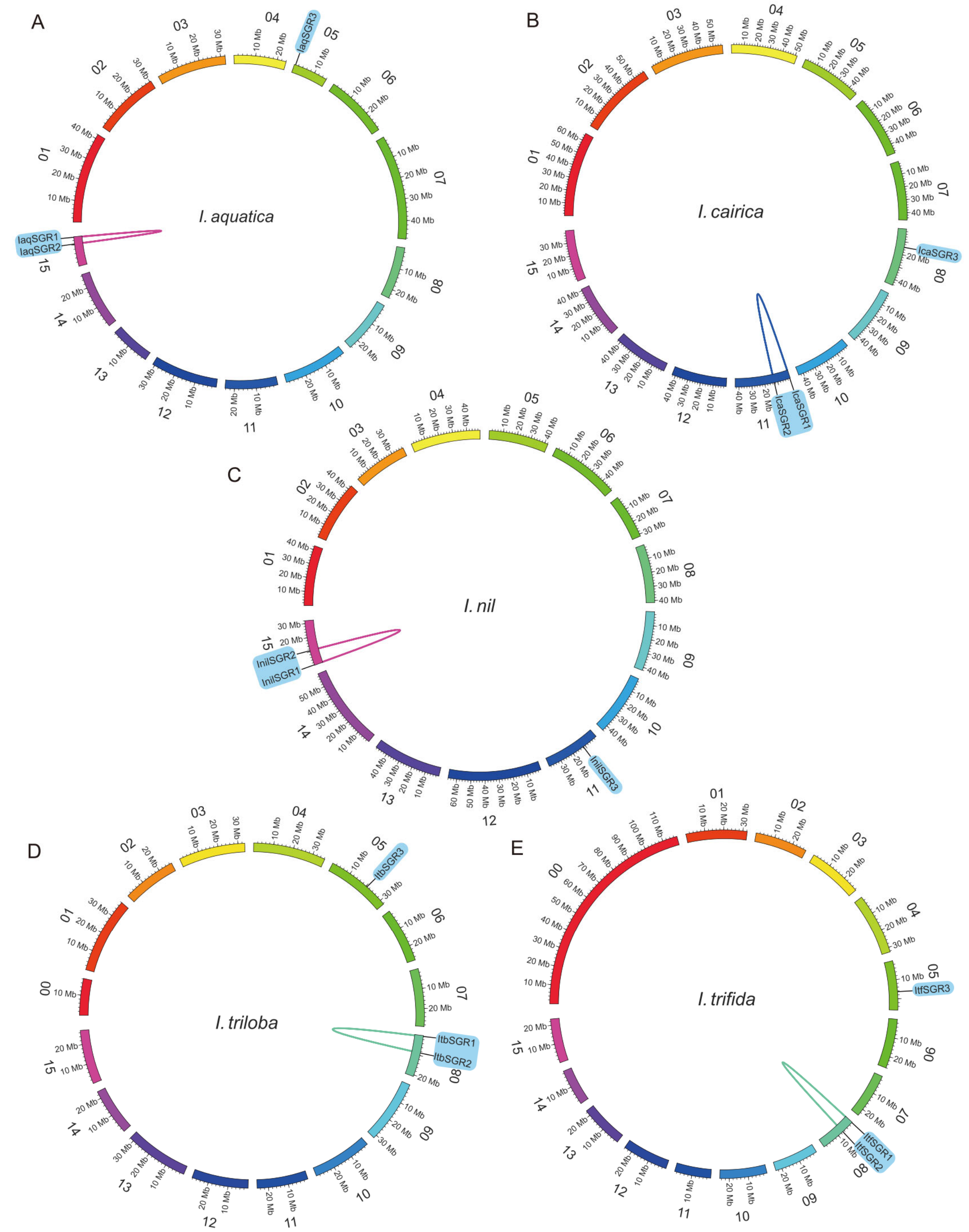

Figure S1. Intraspecific collinearity analysis of *SGRs* in five *Ipomoea* species.

(A-E) Intraspecific collinearity analysis of *I. aquatica*, *I. cairica*, *I. nil*, *I. triloba*, and *I. trifida.*The curves denote the collinearity relationships of *SGRs* in the different species.
